# Transcriptome Analysis of Differentially Expressed Genes Relevant to Variegation in Peach Flowers

**DOI:** 10.1371/journal.pone.0090842

**Published:** 2014-03-06

**Authors:** Yingnan Chen, Yan Mao, Hailin Liu, Faxin Yu, Shuxian Li, Tongming Yin

**Affiliations:** 1 The Southern Modern Forestry Collaborative Innovation Center, Nanjing Forestry University, Nanjing, Jiangsu, China; 2 Institute of Biology and Resources, Jiangxi Academy of Sciences, Nanchang, Jiangxi, China; Nazarbayev University, Kazakhstan

## Abstract

**Background:**

Variegation in flower color is commonly observed in many plant species and also occurs on ornamental peaches (*Prunus persica* f. *versicolor* [Sieb.] Voss). Variegated plants are highly valuable in the floricultural market. To gain a global perspective on genes differentially expressed in variegated peach flowers, we performed large-scale transcriptome sequencing of white and red petals separately collected from a variegated peach tree.

**Results:**

A total of 1,556,597 high-quality reads were obtained, with an average read length of 445 bp. The ESTs were assembled into 16,530 contigs and 42,050 singletons. The resulting unigenes covered about 60% of total predicted genes in the peach genome. These unigenes were further subjected to functional annotation and biochemical pathway analysis. Digital expression analysis identified a total of 514 genes differentially expressed between red and white flower petals. Since peach flower coloration is determined by the expression and regulation of structural genes relevant to flavonoid biosynthesis, a detailed examination detected four key structural genes, including *C4H*, *CHS*, *CHI* and *F3H*, expressed at a significantly higher level in red than in white petal. Except for the structural genes, we also detected 11 differentially expressed regulatory genes relating to flavonoid biosynthesis. Using the differentially expressed structural genes as the test objects, we validated the digital expression results by using quantitative real-time PCR, and the differential expression of *C4H*, *CHS* and *F3H* were confirmed.

**Conclusion:**

In this study, we generated a large EST collection from flower petals of a variegated peach. By digital expression analysis, we identified an informative list of candidate genes associated with variegation in peach flowers, which offered a unique opportunity to uncover the genetic mechanisms underlying flower color variegation.

## Background

Flower color is of paramount importance in plant biology [1]. Three major groups of pigments–betalains, carotenoids and flavonoids–are responsible for the attractive natural display of flower colors [2], [3]. Flavonoids, particularly anthocyanidins, are the most common flower pigments, and contribute to a wide range of colors, from pale yellow to red, purple and blue [4]. To date, most enzymes involved in the anthocyanin biosynthetic pathway have been identified in various plant species [5]–[7]. All anthocyanidins are derived from a general phenylpropanoid pathway that converts the aromatic amino acid phenylalanine to anthocyanidins through a series of enzyme-catalyzed reactions [8]–[12]. During the past few decades, much of the molecular information available on the regulation and biosynthesis of flower pigments has been derived from studies performed in model systems such as *Zea mays* L. (maize) [13], [14], *Arabidopsis* [6], [15], petunia [16], [17] and snapdragon [3], [15]. An increasing number of non-classical plants, however, are providing unique insights into molecular mechanisms involved in flower pigment formation, leading to further understanding of how flower color varies among wild species [18]–[20].

**Figure 1:**
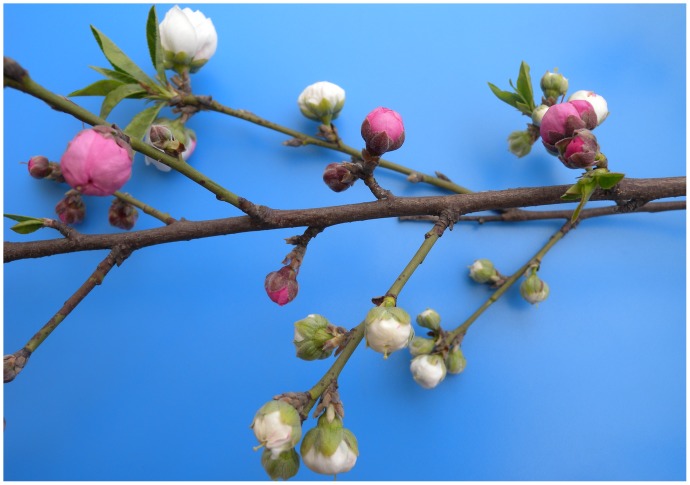
Sampling stage of variegated flower petals.

Ornamental peach, a member of the *Rosaceae* family, is an important horticultural tree. *Prunus persica* f. *versicolor* (Sieb.) Voss, one of the main varieties, is characterized by the presence of chimeric flowers (Figure 1). Because variegation in flowers often attracts consumer attention, variegated plants are generally of high value in the ornamental market. This unstable phenotype has been observed in natural populations of petunia, snapdragon, morning glory, azalea and other plant species [21]. Flower variegation is usually due to the presence of a group of colored cells descended from a single ancestral cell in which a somatic mutation from the recessive white to the pigmented revertant allele has occurred. Somatic mutation frequency and timing during petal development determine variegation patterns [22].

Molecular mechanisms of flower variegation have been investigated in various plant species [22]–[25]. Some of this variegation is caused by transposable element insertion into structural genes associated with the anthocyanin synthetic pathway [26]–[28]. Because of the complexity of the flavonoid biosynthetic pathway, however, the exact genetic mechanisms underlying the generation of flower pigmentation chimeras may differ among species. Chaparro et al. [29] studied variegation in anthocyanin production in both vegetative and reproductive tissues of the peach cultivar Pillar; they verified that this phenotype is heritable, although the degree of variegation differed according to the genetic background of outcross progeny. The genetic mechanisms in peach responsible for this unstable phenotype have not yet been elucidated. The whole peach genome has been sequenced [30], and abundant transcriptome sequences are available to the public [31]. The reference genome and large EST collection enhance our ability to align sequences, identify genes, and characterize transcriptomes, thereby facilitating identification of the genetic basis of variegated pigmentation in peach.

To understand the mechanism of variegated pigmentation, detection of differentially expressed genes from different-colored flowers is essential. Transcriptome sequencing is an efficient way to measure transcriptome composition and uncover differentially expressed genes [32]–[34]. Many studies using high-throughput next-generation sequencing technology have surveyed the complex transcriptomes of various plants including *Arabidopsis thaliana* [35], *Digitalis purpurea* [36], *Carthamus tinctorius* [37], *Persea americana* [38] and *Salix suchowensis* [39]. In this study, transcriptomes of white and red flower petals sampled from a *Prunus persica* f. *versicolor* individual were sequenced using a 454 GS-FLX sequencer. By analyzing the data with various bioinformatics tools, we aimed to discover candidate genes involved in peach flower variegation.

## Materials and Methods

### Plant Material

We separately collected petals of expanded but unflushed white and red variegated flower buds (Figure 1) from a tree of *Prunus persica* f. *versicolor* in Nanjing Lovers Garden, Jiangsu, China in March 2012. Petals were immediately frozen in liquid nitrogen and stored at −80°C until RNA extraction. The field studies did not involve any endangered or protected species, and sample collection was authorized by the administration office of Nanjing Lovers Garden.

### RNA Extraction and cDNA Synthesis

Total RNA was extracted separately from white and red petals using the CTAB method [39]. RNA integrity was confirmed by 1% agarose gel electrophoresis. After digestion with DNase (Takara) at 37°C for 30 min to remove DNA residues, RNA concentration was determined using a Nanodrop spectrophotometer (Thermo). mRNA was then purified from total RNA using an Oligotex mRNA midi kit (Qiagen), with its quality assessed using an Agilent Technologies 2100 Bioanalyzer (Agilent). cDNA synthesis was performed using the 454 cDNA amplification technique with a cDNA Synthesis System kit (Roche) following the manufacturer’s protocol.

### Sequencing Library Construction and 454 Sequencing

Sequencing libraries were separately constructed for white and red petals using a Rapid Library Prep kit (Roche). Quality of sequencing libraries was checked using an Agilent 2100 Bioanalyzer. Approximately 2.1 million beads per flower color were separately loaded onto two sections of a pico-titer plate. A sequencing run was carried out on a Roche 454 GS FLX sequencer at Nanjing Forestry University. All ESTs in this study were deposited in NCBI with an accession number SRR1037160.

### EST Sequence Processing and Assembly

Raw 454 sequence files in SFF format were base-called using the Pyrobayes base caller [40]. In addition, 78,689 peach ESTs were downloaded from GenBank in February 2012. The 454 sequencing data and GenBank ESTs were processed with GS FLX v2.0.01 software (454 Life Sciences, Roche) to remove low-quality and adaptor sequences. To remove possible contamination, the resulting high-quality 454 and GenBank sequences were screened against the NCBI UniVec database, *E. coli* genome sequences, and peach ribosomal RNA and chloroplast genome sequences. Sequences shorter than 50 bp were discarded before assembly. Finally, the processed 454 and GenBank sequences were assembled into putative transcripts (including contigs and singletons) using the 454 assembly program Newbler v2.7 with the following overlap detection settings: seed step, 12; seed length, 16; seed count, 1; minimum overlap length, 40; minimum overlap identity, 95%; alignment identity score, 2; alignment difference score, −3. We parsed the 454 ReadStatus.txt file to identify singletons, which were unassembled reads. The contig and singleton files were used to generate a unigene file.

### Mapping Unigenes to Peach Genome Predicted Genes

The generated unigenes were aligned to predicted genes of the peach genome using BLAST [41]. Based on the International Peach Genome Initiative (IPGI; http://www.rosaceae.org/species/prunus/prunus_persica), there are 27,864 predicted genes in the peach genome. Because the number of obtained unigenes was much greater than the number of predicted genes, it is possible that different unigenes were segments of the same predicted gene. In this study, unigenes mapping to the same predicted gene were integrated into one unique gene, which was used to collect transcript count information for detection of differentially expressed genes in white and red flower petals.

### Gene Annotation and Pathway Prediction

Unigene annotation was performed by BLASTX analysis [42] against peach protein (http://www.phytozome.com/peach.php; v2.0), NCBI non-redundant protein (nr) and UniProt databases using a cutoff *E*-value of 10^−5^. Gene Ontology (GO) terms were assigned to each unigene based on GO annotations of its corresponding homologs in the UniProt database [43], and, using interpro2go and pfam2go mapping files available on the GO website (http://www.geneontology.org), their corresponding InterPro and Pfam domains. GO mapping results were further plotted by uploading the GO list file to the Web Gene Ontology Annotation Plot (WEGO) website (http://wego.genomics.org.cn/cgi-bin/wego/index.pl). The detailed annotation was then used to retrieve keywords for identification of flower pigmentation-related genes.

The KEGG Automatic Annotation Server (KAAS; http://www.genome.jp/tools/kaas/) was employed to perform metabolic pathway mapping [44], [45]. KAAS assigned each peach gene a KEGG Orthology (KO) number, which was then used for mapping to a KEGG reference metabolic pathway.

### Differentially Expressed Genes between White and Red Petals

Following cDNA sequence assembly and gene prediction, transcript count information was collected for sequences corresponding to each predicted gene associated with a flower petal color. To obtain relative expression levels in each sample, transcript counts were normalized to the total number of produced transcripts per sample. Significance of gene differential expression level was assessed using R [46], χ^2^ and Fisher exact tests as implemented in the publicly available web tool IDEG6 (http://telethon.bio.unipd.it/bioinfo/IDEG6/) [47]. A gene was considered to be differentially expressed when results from the above tests were all significant at a level of *P*≤0.0001.

### Quantitative Real-time PCR Analysis

Total RNA was extracted from red and white flower petals as described above. Approximately 2 µg of total RNA per sample was treated with DNaseI (Takara), and then subjected to reverse transcript to cDNA using reverse transcription system (Promega). The quantitative real-time PCR (qRT-PCR) was performed using Applied Biosystems 7500 Real-time PCR system (Applied Biosystems) with SYBR Premix Ex Taq (Takara). Each reaction contained 2 µL the first-strand cDNA as template, in a total volume of 20 µL reaction mixture. The amplification program was performed as 95°C 30 s followed by 95°C for 5 s and 60°C for 34 s (40 cycles). Gene-specific primers, shown in Table S3, were used for detecting the relative quantification of each gene. qRT-PCR expression levels were compared based on the mean of three independent experimental repeats. Calculation of relative expression level was performed using the 2^–ΔΔCT^ method [48]. *TEF2* was used as an internal control for normalization [49].

## Results

### Transcriptome Sequencing and Assembly

**Figure 2:**
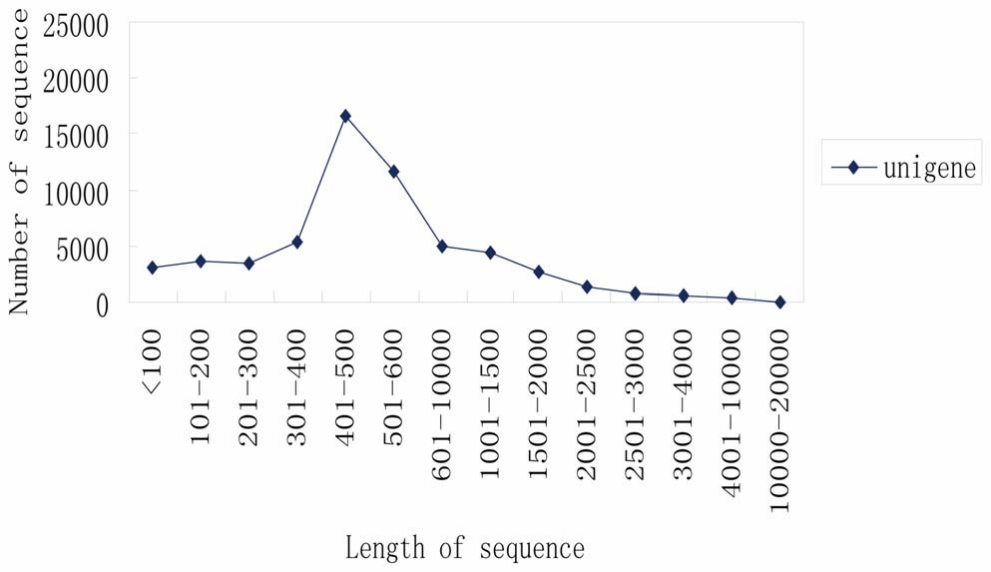
Length distributions of peach unigenes.

**Figure 3:**
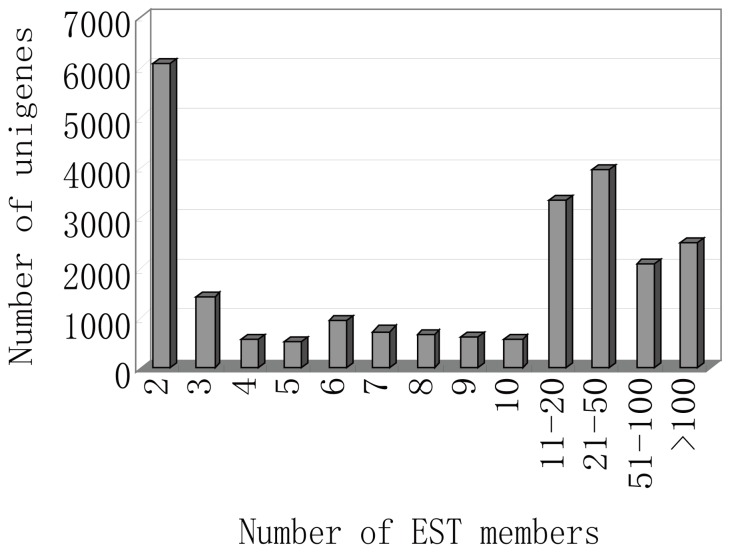
Distribution of ESTs in peach unigenes.

A half 454 GS-FLX run was performed for each sample, resulting in the generation of 1,556,684 reads. After quality control, 1,556,597 reads with an average length of l, 445 bp remained: 837,041 from white petals and 719,556 from red petals (Table 1). These reads, together with 76,822 high-quality GenBank ESTs, were subjected to assembly analysis. Sequence assembly yielded 58,580 unigenes comprising 16,530 contigs and 42,050 singletons. Contigs and singletons had average lengths of 1,555 bp and 364 bp, respectively (Table 2). A plot of unigene length distribution revealed that most unigenes (82.8%) were longer than 300 bp (Figure 2). From our EST collection, we were able to identify a number of highly expressed unigenes in peach flowers (Figure 3). Approximately 12,589 unigenes were assembled from more than 10 EST reads; these unigenes (∼21% of all unigenes) corresponded to ∼95% of total EST reads.

**Table 1:** Statistics for peach ESTs generated by 454 GS-FLX sequencing.

	Red petals	White petals	Total
**Number of reads**	719,556	837,041	1,556,597
**Average read length (bp)**	443.3	448	445.8
**Total bases (bp)**	318,983,491	374,962,455	693,945,946
**Number of reads in contigs**	675,990	783,159	1,459,149
**Number of reads as singletons**	28,444	35,141	63,585

**Table 2:** Statistics for assembled peach unigenes.

	Singleton	Contig	Unigene(cluster)
**Number of sequences**	42,050	16,530	58,580
**Average read length (bp)**	364	1,555	699
**Total bases (bp)**	26,469,716	27,763,950	40,933,465
**Number of unigenes mapped to peach genome predicted genes**	25,042	15,645	40,687
**Number of unigenes unmapped to peach genome predicted genes**	17008	885	17893

### Mapping Unigenes to Predicted Genes in the Peach Genome

**Figure 4:**
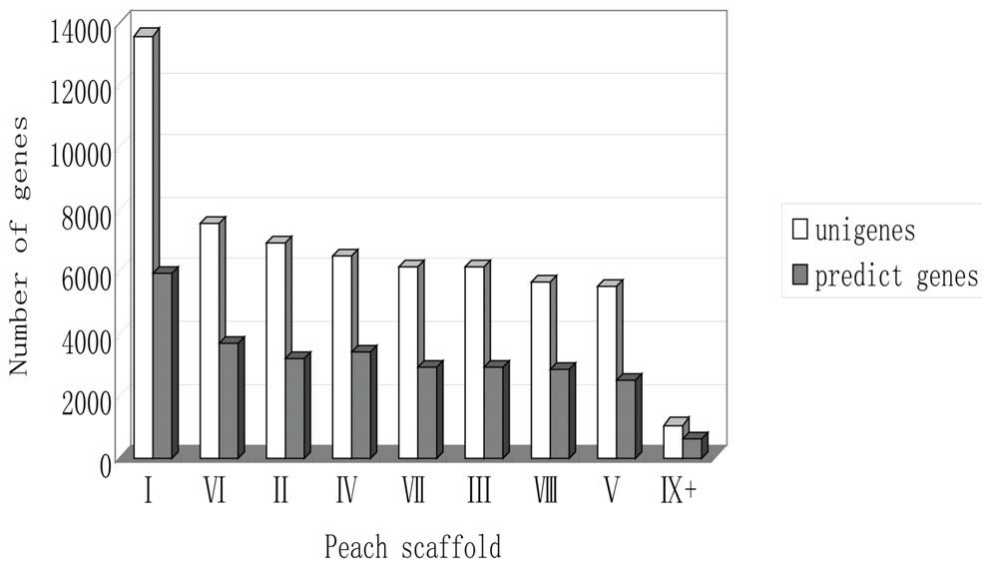
Distribution of unigenes and predicted genes allocated to each peach genome scaffold. Roman numerals I–VIII correspond to the eight large scaffolds of the peach genome (v1.0; IPGI), and are ordered along the x-axis with the number of mapped unigenes on each scaffold. IX+ refers to all remaining small scaffolds of the peach genome.

Using the peach genome (v1.0; IPGI), approximately 70% (40,687) of obtained unigenes could be mapped to predicted genes of *P. persica*. Among these, 15,645 were contigs (95% of all contigs) and 25,042 were singletons (60% of singletons). The mapped unigenes were aligned to 16,733 predicted genes, corresponding to approximately 60% of the 27,864 predicted genes in the peach genome. The remaining 17,893 unigenes could not be mapped to any predicted genes. Among the unmappable unigenes, 885 were contigs and 17,008 were singletons (Table 2). We further examined the unmappable unigenes, and found that about 20.6% had no significant matches with plant sequences. In total, alignment of unigenes to predicted *P. persica* genes generated 34,626 unique genes: 16,733 identified as peach genome predicted genes and 17,893 unmappable unigenes. When unigenes were mapped to different scaffolds of the peach genome (http://www.rosaceae.org/species/prunus/prunus_persica), the number of mapped unigenes was found to be highly correlated with the number of predicted genes on each scaffold (paired t-test, R^2^ = 0.992) (Figure 4). The number of expressed genes was thus proportional to the number of predicted genes allocated to each scaffold.

### Functional Annotation of Peach Transcriptomes

Based on alignments of unigenes to peach genome predicted genes, 34,626 unique genes were identified. To infer putative functions of these unique genes, we blasted their sequences against the GenBank nr database using a significance cutoff of *E* ≤10^−5^. The analysis indicated that 21,663 unique genes (63%) had significant matches to the nr database, among which 15,846 were peach genome predicted genes (94.7% of the 16,733 unigene-matched predicted genes) and 5,817 were unmappable unigenes (32% of all unmappable unigenes). Most of the unmappable unigenes were singletons (95.1%), which were much shorter than contigs (364 bp vs. 1,555 bp on average). In general, the longer the sequence is, the greater the chance of annotation, and thus the more number of GO terms that can be recovered [50]. This suggests that the inability to assign putative functions to most unmappable unigenes was due to the absence of conserved functional domains in short sequences.

**Figure 5:**
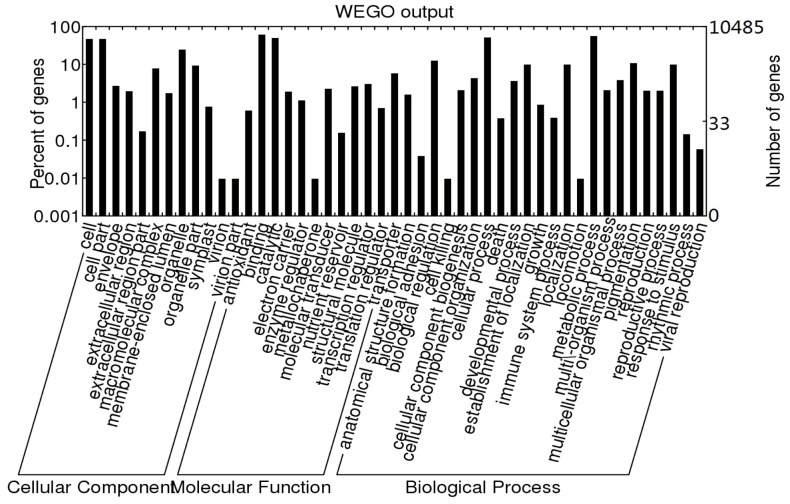
Number of peach unigenes in each functional category. Peach unigenes were classified into different functional groups based on a set of plant-specific GO slims within cellular component, molecular function and biological process categories.

Gene Ontology (GO) describes gene products in terms of their associated molecular functions, biological processes and cellular components. To assign putative functional roles to the obtained unique genes, GO terms were assigned based on sequence similarities to known GO-annotated proteins (and their InterPro and Pfam domains) in the UniProt database. As a result, 10,485 unique genes were assigned at least one GO term. Among GO terms, 896 were in the biological process category, 606 in the molecular function category, and 607 in the cellular component category (Figure 5). These unique genes were further displayed using a set of GO slims, which are a list of high-level GO terms providing a broad overview of ontology content (http://www.geneontology.org/GO.slims.shtml). Figure 5 displays the functional classification of peach unique genes into plant-specific GO slims within cellular component, molecular function and biological process categories. Genes involved in cell, cell part, binding, catalytic, cellular process and metabolic process categories were the highest-represented groups, indicating that flower buds were undergoing rapid growth and carrying out intensive metabolic activities. In the biological process category, it is noteworthy that genes involved in pigmentation were also highly represented, indicating active pigmentation activities.

### Biochemical Pathways

KEGG, an alternative functional gene annotation system, performs assignments based on Enzyme Commission (EC) numbers of genes associated with biochemical pathways. To further demonstrate the usefulness of the generated peach ESTs for discovering flower pigmentation-related genes, we identified biochemical pathways represented by our EST collection. Annotations of peach unique genes were fed into the Pathway Tools program (KAAS; http://www.genome.jp/tools/kaas/). This process predicted 229 pathways represented by a total of 5,345 unique genes. Of these KEGG-annotated genes, 2,425 (45.4%) were involved in metabolism, 1,186 (22.2%) in genetic information processing, 346 (6.5%) in environmental information processing, 569 (10.6%) in cellular processes and 792 (14.8%) in organism systems (Table S1). Among genes involved in metabolism, 14 genes were found to encode key enzymes involved in flavonoid biosynthesis. We mapped and highlighted these genes onto the “flavonoid biosynthesis” pathway (Figure S1), which demonstrated that most of the key enzymes in this pathway were covered by our sequence data. We also detected three genes involved in flavone and flavonol biosynthesis; they were mapped and highlighted onto the “flavone and flavonol biosynthesis” pathway (Figure S2). In contrast to the flavonoid biosynthesis pathway, only a small proportion of flavone and flavonol biosynthetic pathway key enzymes were represented by our sequence data.

### Identification of Differentially Expressed Genes in White and Red Flower Petals

In this study, we obtained 16,530 contigs and 42,050 singletons, among which 15,645 contigs and 25,042 singletons were mapped to 16,733 peach predicted genes. There were 855 unmapped contigs and 17,008 unmapped singletons. The purpose of this study was to uncover genes differentially expressed between flower petal colors. The 17,008 unmapped singletons were less closely related to peach genome predicted genes and were not present in sufficient quantities for statistical analysis [50]; they were consequently excluded from the differentially expressed gene analysis. Number of reads was obtained for each gene using a custom PERL script. The digital expression profiling analysis identified 514 genes differentially expressed between red and white flower petals, with *P*<0.0001 for all employed statistics (Table S2); 367 of these genes showed significantly higher expression in red flower petals, whereas 147 exhibited significantly higher expression in white petals.

In peach, the flavonoid biosynthetic pathway leads to production of colored pigments. We therefore specifically examined expression of flavonoid structural genes, and identified four differentially expressed genes, all highly expressed in red petals. These genes encode the enzymes chalcone and stilbene synthase (CHS), chalcone-flavanone isomerase (CHI), cinnamate-4-hydroxylase (C4H) and flavanone 3-hydroxylase (F3H). Functionally, C4H catalyzes the incorporation of a 4′-hydroxyl group into cinnamate during p-coumarate formation. CHS condenses one molecule of p-coumaroyl-CoA with three molecules of malonyl-CoA to produce the chalcone tetrahydroxychalcone. (Chalcone is the precursor for all classes of flavonoids, including flavones, flavonols, flavandiols, flavan-4-ols, condensed tannins, isoflavonoids and anthocyanins.) CHI is responsible for the conversion of tetrahydroxychalcone to naringenin, and F3H catalyzes the formation of DHK from naringenin (Figure S3). DHK can be further hydroxylated to form dihydroquercetin (DHQ) and dihydromyricetin (DHM). DHK, DHQ and DHM subsequently lead synthetic branches producing pelargonidin-based (orange to red), cyanidin-based (red to magenta) and delphinidin-based (purple) pigments, respectively. Interestingly, the four differentially expressed genes all mapped to early committed steps on the flavonoid biosynthetic pathway before the formation of DHK (Figure S3). From 454 sequencing, absolute read numbers of *C4H*, *CHS*, *CHI* and *F3H* in red vs. white flower petals were 146∶54, 4050∶1448, 122∶14 and 26∶1, respectively. After normalization, expression levels of *C4H*, *CHS*, *CHI* and *F3H* were 3.1-, 3.3-, 10.1- and 30.2-fold higher, respectively, in red flower petals than in white ones.

In the flavonoid biosynthetic pathway, transcription levels of flavonoid biosynthesis genes are regulated by various TFs. TFs related to flavonoid biosynthesis can be divided into three classes: MYB, basic-Helix-Loop-Helix (bHLH) and WD40 [15], [51], [52]. Apart from the structural genes, we further examined expression levels of these three TF classes. We detected 11 TFs differentially expressed between red and white petals, including 3 bHLHs, 4 MYBs and 4 WD40s. Nine of these TFs were highly expressed in red petals, and two were highly expressed in white petals (Table 3). These DNA-binding proteins interact with promoter regions of target genes and regulate the initiation rate of mRNA synthesis.

**Table 3:** Differentially expressed structural and regulatory genes related to flavonoid pathways in variegated peach flowers.

ID	Function distribution	Highly expressed tissue
ppa025745m	Chalcone and stilbene synthase (CHS) family protein	red
ppa011276m	Chalcone-flavanone isomerase (CHI) family protein	red
ppa004544m	cinnamate-4-hydroxylase (C4H)	red
ppa007636m	flavanone 3-hydroxylase (F3H)	red
ppa009757m	basic helix-loop-helix (bHLH) DNA-binding superfamily protein	red
ppa003543m	basic helix-loop-helix (bHLH) DNA-binding superfamily protein	red
ppa013751m	ILI1 binding bHLH 1	red
ppa010069m	myb domain protein 113	red
ppa011751m	myb domain protein 24	red
ppa010277m	myb domain protein 4	red
ppa007222m	myb-like transcription factor family protein	white
ppa005673m	Transducin family protein/WD-4 repeat family protein	red
ppa005800m	Transducin family protein/WD-4 repeat family protein	red
ppa002072m	Transducin/WD4 repeat-like superfamily protein	red
ppa014936m	Transducin/WD4 repeat-like superfamily protein	white

**Figure 6:**
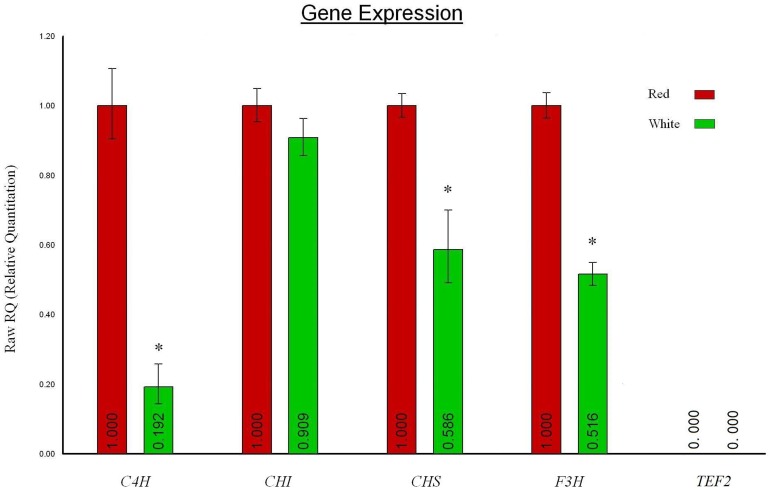
qRT-PCR analysis of *C4H*, *CHS*, *CHI* and *F3H* gene expression in white and red flower petals. *TEF2* was used as an internal control for normalization. qRT-PCR data calculated with the 2^−ΔΔCt^ method [48]. The expression level of each gene in red flower petals was arbitrarily set as 1 and its corresponding transcript level in white flower petals was calibrated against red one. Error bars represent standard error for three independent experimental replicates. *, p<0.05, as determined by one-way ANOVA.

Using the differentially expressed structural genes as the test objects, we further validated the digital expression profiling by qRT-PCR technology. Statistically, *C4H*, *CHS*, and *F3H* expressed significantly higher in red flower petals than in white ones, with p<0.05 (Figure 6). Thus, the significant difference in expression level of these three structural genes was confirmed by both of the analytical techniques. Whereas the expression level of *CHI* was not statistically different between colors. The comparative transcription level of this gene was found to be only slightly higher in red than in white petals by qRT-PCR analysis (Figure 6). Nevertheless, in general, the qRT-PCR results were in rough accordance with the electronic data of gene expression analysis.

## Discussion

Approximately 60% of total predicted genes in the peach genome were covered by the unigenes obtained in this study. In *Arabidopsis*, approximately 55–67% of genes are expressed in a single tissue based on microarray analysis [53]. In human and mouse, around 60–70% of genes are expressed in a specific tissue [54]. Transcriptomic studies have revealed that about 64%, 66% and 68% of genesare expressed in whole flower tissues of cucumber [55], willow [39] and peach [31], respectively. In our study, we sequenced genes expressed in flower petals, not entire flowers. Our EST collection, designed to capture the majority of genes expressed in peach flower petals, would thus be expected to encompass fewer expressed genes. Because more transcript sequences were generated in peach (1.5 million) than in willow (1.2 million) [39] or cucumber (0.35 million) [55], the sequence depth of genes covered by our EST dataset should therefore be higher. We thus obtained a reliable dataset to explore differentially expressed genes relevant to variegation in peach flowers. Most differentially expressed genes that have currently been revealed by transcriptome sequencing are derived from studies performed with tissues of different genotypes [39], [55], [56]. In contrast, ESTs generated in our study are collections of expressed genes of the same genotype. Our approach is thus appropriate for identification of differentially expressed genes relevant to the focal phenotype.

The primary pigments related to flower color are anthocyanins, which contribute to a variety of colors, such as red, pink and blue [4]. Biochemical pathway mapping revealed that most of the structural genes in the anthocyanin biosynthetic pathway were covered by our sequence data (Figure S1), and we detected four structural genes highly expressed in red petals. In addition to anthocyanins, some co-pigments, such as flavones and flavonols, can change flower color hues. Only a few structural genes (Figure S2) involved in flavone and flavonol biosynthesis were captured, however, and none of them were differentially expressed between red and white petals. These results suggest that peach flower variegation is linked mainly to anthocyanin biosynthesis, with flavones and flavonols having a limited role.

With respect to enzymatic genes involved in the anthocyanin biosynthetic pathway, *C4H*, *CHS* and *F3H* were confirmed to express at a significantly higher level in red petals than in white petals by both the transcriptome profiling and qRT-PCR analysis. These genes were associated with committed steps before the formation of DHK (Figure S3). Because red flower color in peach is related to the synthesis of pelargonidin-based (orange to red) and cyanidin-based (red to magenta) pigments, our results suggest that the low *C4H*, *CHS* and *F3H* expression levels in white petals reduce DHK formation, thereby inhibiting pelargonidin and cyanidin production. In contrast, the high expression levels of these genes observed in red petals ensure sufficient anthocyanin yields to make flowers red. In this experiment, none of the structural genes downstream of DHK (Figure S3) were found to be significantly differentially expressed between white and red petals. It can thus be concluded that variegation in peach flowers is related primarily to structural genes upstream of DHK in the anthocyanin synthetic pathway, and is less likely affected by genes downstream.

In nature, white is the most abundantly occurring flower color, and can also be artificially produced from colored flowers. The first successful reversion to white flowers was achieved by suppressing *CHS* in tobacco and petunia [57]. Suppression of structural genes in the anthocyanin synthetic pathway has subsequently proven useful for modifying colored flowers to white. Studies have shown that transgenic plants carrying single gene constructs of *CHS* [58], [59] or *F3H* [60] can all exhibit white or faint-colored flowers.

Final anthocyanin concentrations in plant cells are not determined solely by structural gene expression levels; some regulatory genes are clearly also involved in control of flavonoid biosynthesis gene expression. These regulatory genes, typically specific TFs, influence anthocyanin biosynthesis intensity and pattern and generally control expression of many different structural genes [21]. Three classes of TFs–bHLH, MYB and WD40–have been found to be related to flavonoid biosynthesis [15], [51], [52]. In maize, an MYB-related protein and a bHLH-containing protein have been shown to interact to activate genes in the anthocyanin biosynthetic pathway [61]. In this study, we detected 11 differentially expressed TFs from the three flavonoid biosynthesis-related classes. The high *C4H*, *CHS* and *F3H* expression levels observed in red peach flower petals may be due to regulation by one or more of these TFs. Although the exact regulatory TFs remain unknown, this study provides an informative list of candidates.

In peach flowers, different variegation patterns are observed: some occur among different flowers on the same branch, while others occur within the same flower (Figure S4). Much genetic evidence supports the hypothesis that flower variegation is caused by transposable elements inserted into structural genes or regulatory elements related to anthocyanin synthesis [26]–[28]. The different transcript levels detected in this study for structural genes and TFs might be due to insertions of transposable elements. Examination of candidate DNA sequences for transposable element insertions is needed to determine the mechanisms of peach flower variegation. Although transposable elements cannot be detected based solely on transcriptome sequencing, our study has provided some novel insights into the molecular mechanisms underlying variegation in peach flowers.

## Conclusions

Flower color is one of the most attractive characteristics of ornamental plants, and variegated plants are highly valuable in the floricultural market. In this study, we generated a large EST collection from flower petals of a variegated peach. Based on the digital expression analysis, a total of 514 genes were identified to differentially express between red and white flower petals. The red and white petals were collected from the same tree, and they were in the same developmental stage. Moreover, flower coloration was specifically connected with the flavonoid biosynthetic pathway. All these conditions enabled us to narrow down the considerable number of differentially expressed genes to a small number of candidates, which warranted further investigation. Finally, three key structural genes in the anthocyanin biosynthesis pathway were confirmed to express significantly different between colors, and we also detected 11 differentially expressed TFs related to anthocyanin biosynthesis. Our results provide critical information for uncovering candidate genes associated with variegation in peach flowers. We believe this transcriptome dataset will continue to provide unique insights into the molecular mechanisms controlling variegated flower pigmentation, and will eventually help the molecular engineering of variegated plants.

## Supporting Information

Figure S1 **Schematic representation of the flavonoid biosynthesis pathway.** Each box represents a structural gene encoding a key enzyme involved in the flavonoid biosynthesis pathway. Numbers in each box are EC codes of each gene. Genes in red and green boxes represent those captured by our sequence data, with red boxes indicating genes expressed significantly higher in red than in white petals, and green boxes corresponding to genes with insignificant expression differences between colors. Uncolored boxes indicate uncaptured genes. EC code definitions can be found at: http://www.genome.jp/kegg-bin/show_pathway?map00941.
(JPG)

Figure S2 **Schematic representation of the flavone and flavonol biosynthesis pathway.** Each box represents a structural gene encoding a key enzyme involved in the flavone and flavonol biosynthesis pathway. Numbers in each box are EC codes of each gene. Genes in green boxes represent those captured by our sequence data with insignificant expression differences between colors. Uncolored boxes correspond to uncaptured genes. EC code definitions can be found at: http://www.genome.jp/kegg-bin/show_pathway?map00944.
(JPG)

Figure S3 **Mapping of enzymes coded by differentially expressed structural genes to the flavonoid biosynthetic pathway.** Enzymes corresponding to each of the differentially expressed structural genes are outlined in red.
(JPG)

Figure S4 **Natural occurrence of different flower colors in peach (** ***Prunus persica*** **f.** ***versicolor*** **[Sieb.] Voss).** (A) Flowers having different colors on the same branch. (B) A chimeric flower composed of white and red sections.
(JPG)

Table S1 **Biochemical pathways represented by ESTs from this study.**
(XLS)

Table S2 **Genes differentially expressed between white and red petals from variegated peach flowers in this study.** Structural genes and transcription factors related to flavonoid/anthocyanin biosynthesis are highlighted in red.
(XLS)

Table S3 **Gene-specific primers used in qRT-PCR analysis.** pF represents forward primer and pR represents reverse primer. The primer sequences of *TEF2* were refered to Tong et al. [49].
(DOC)
