# Supplementary figures and images for: Transcriptome Analysis of Differentially Expressed Genes Relevant to Variegation in Peach Flowers
Source: PLoS One. 2014 Mar 6;9(3):e90842. doi: 10.1371/journal.pone.0090842 (PMC3948169; doi:10.1371/journal.pone.0090842)

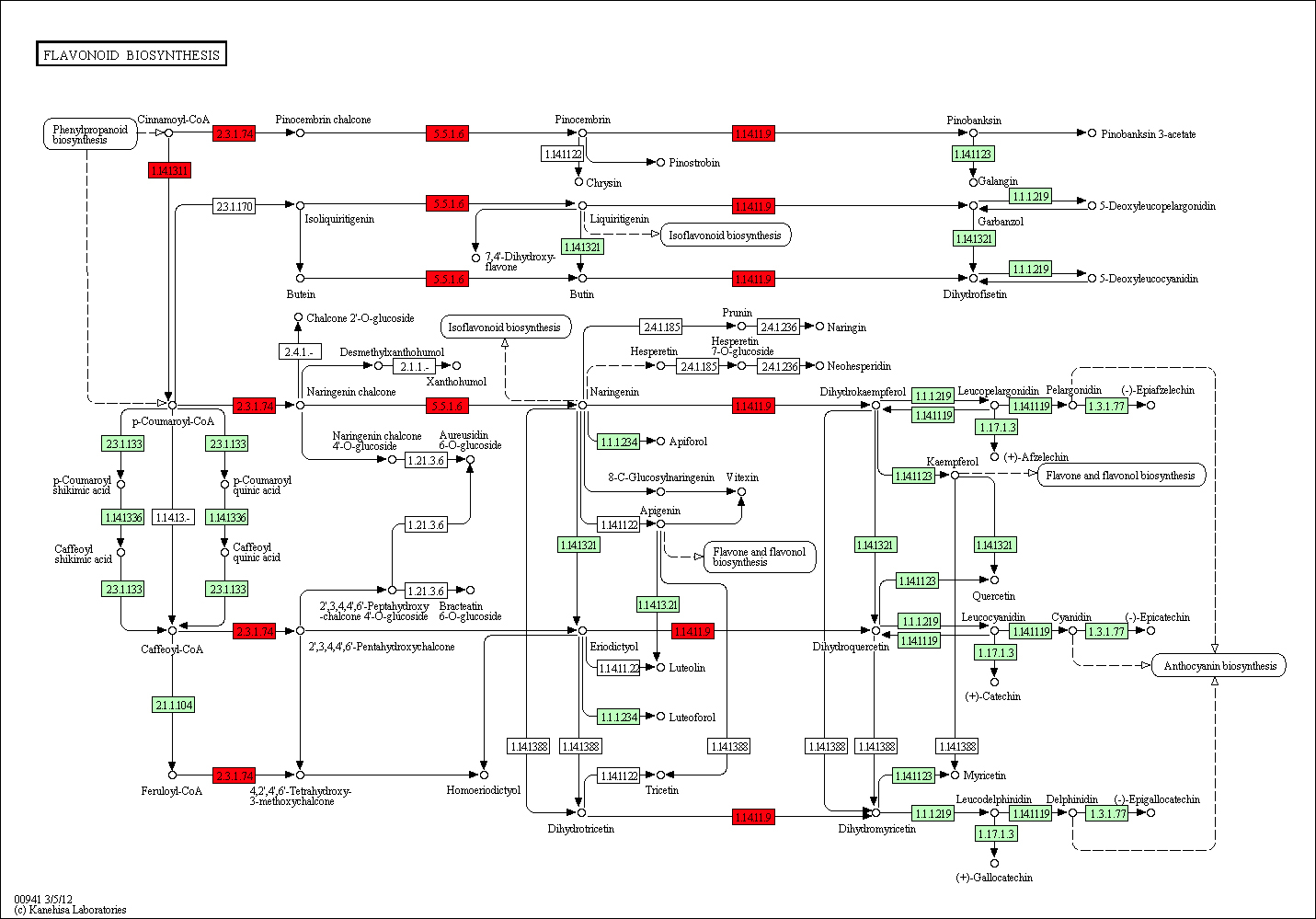

Supplement: Figure S1 — Schematic representation of the flavonoid biosynthesis pathway. Each box represents a structural gene encoding a key enzyme involved in the flavonoid biosynthesis pathway. Numbers in each box are EC codes of each gene. Genes in red and green boxes represent those captured by our sequence data, with red boxes indicating genes expressed significantly higher in red than in white petals, and green boxes corresponding to genes with insignificant expression differences between colors. Uncolored boxes indicate uncaptured genes. EC code definitions can be found at: http://www.genome.jp/kegg-bin/show_pathway?map00941. (JPG) [file pone.0090842.s001.jpg]

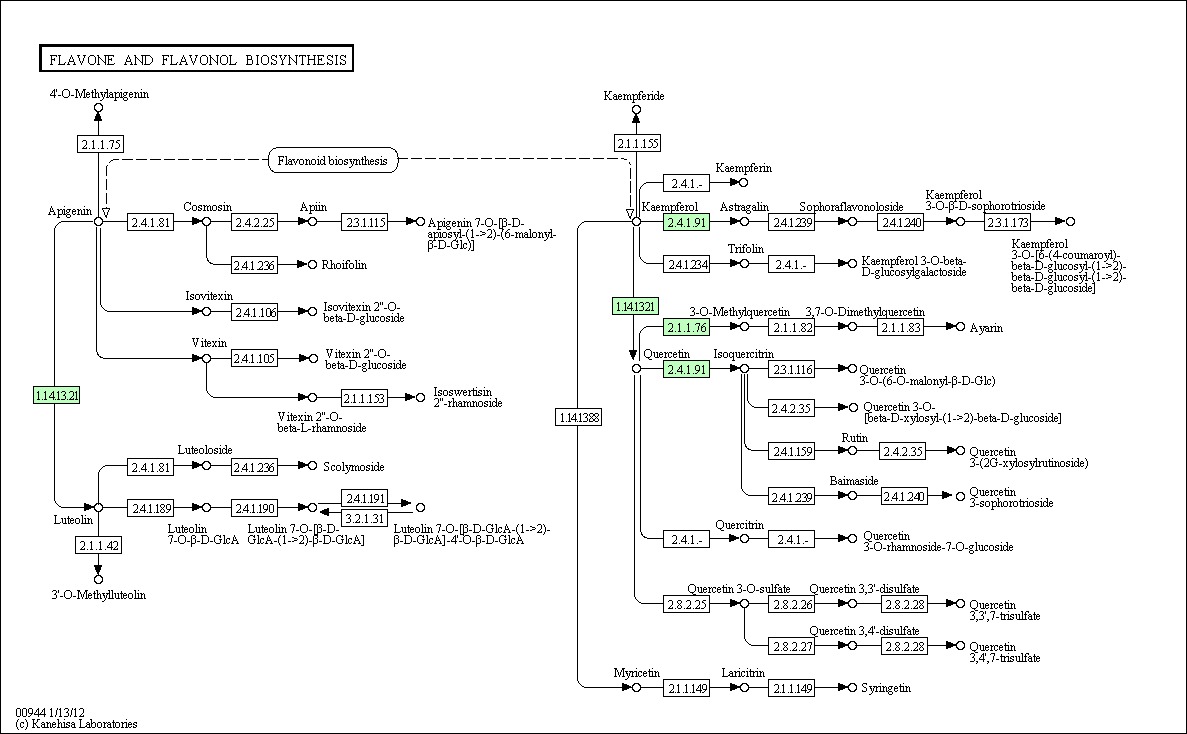

Supplement: Figure S2 — Schematic representation of the flavone and flavonol biosynthesis pathway. Each box represents a structural gene encoding a key enzyme involved in the flavone and flavonol biosynthesis pathway. Numbers in each box are EC codes of each gene. Genes in green boxes represent those captured by our sequence data with insignificant expression differences between colors. Uncolored boxes correspond to uncaptured genes. EC code definitions can be found at: http://www.genome.jp/kegg-bin/show_pathway?map00944. (JPG) [file pone.0090842.s002.jpg]

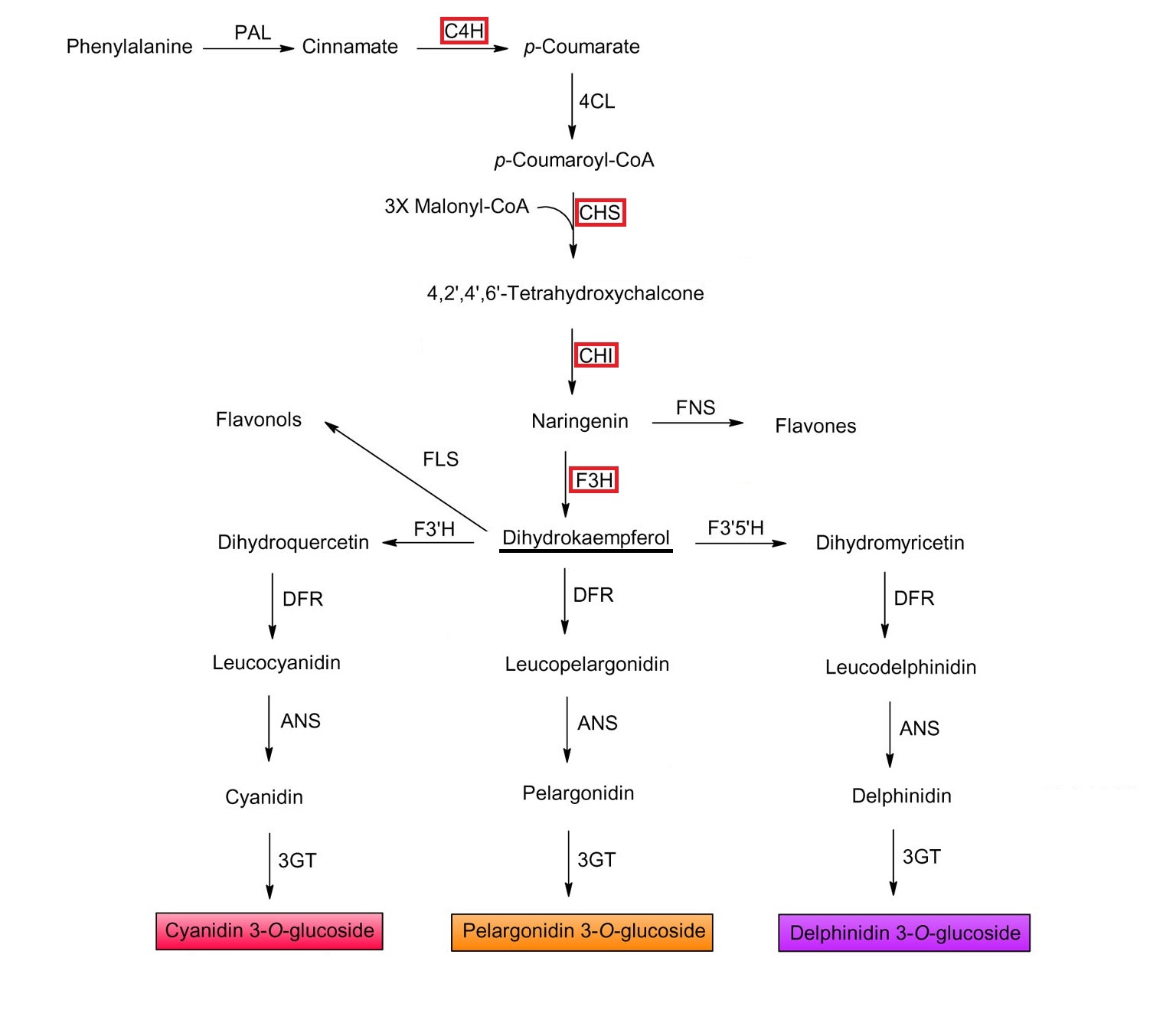

Supplement: Figure S3 — Mapping of enzymes coded by differentially expressed structural genes to the flavonoid biosynthetic pathway. Enzymes corresponding to each of the differentially expressed structural genes are outlined in red. (JPG) [file pone.0090842.s003.jpg]

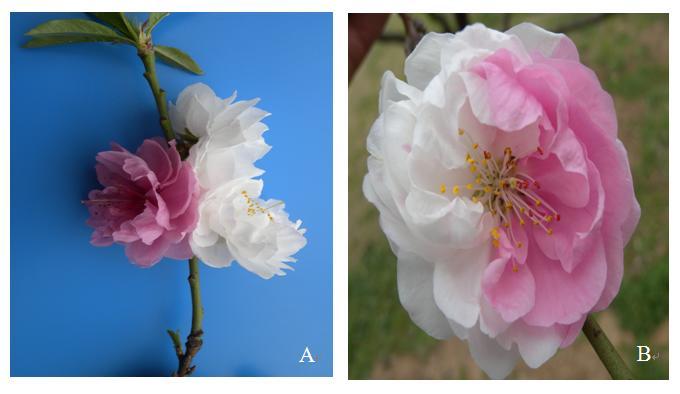

Supplement: Figure S4 — Natural occurrence of different flower colors in peach ( Prunus persica f. versicolor [Sieb.] Voss). (A) Flowers having different colors on the same branch. (B) A chimeric flower composed of white and red sections. (JPG) [file pone.0090842.s004.jpg]
