# Supplementary material for: Transcriptome Analysis of Differentially Expressed Genes Relevant to Variegation in Peach Flowers
Source: PLoS One. 2014 Mar 6;9(3):e90842. doi: 10.1371/journal.pone.0090842 (PMC3948169; doi:10.1371/journal.pone.0090842)
Supplement: Table S3 — Gene-specific primers used in qRT-PCR analysis. pF represents forward primer and pR represents reverse primer. The primer sequences of TEF2 were refered to Tong et al. [49]. (DOC) [file pone.0090842.s007.doc]

| **Gene**  **(Accession No.)** | **Primer sequences (5΄ to 3´)** | **Amplicon Size (bp)** |
| --- | --- | --- |
| *C4H*  (unigene10247) | pF: CCACCCTGAGATCCAAAAGA | 164 |
|  | pR: TCATGTGTGGGACAAGCAAT |  |
| *CHS*  (unigene01417) | pF: CGTCCGCTTTAATTGTTGGT | 221 |
|  | pR: AGTTCCAATCATTATTTATCCC |  |
| *CHI*  (unigene07701) | pF: CTAACCGGCCAGCAATACTC | 239 |
|  | pR: ATTTTCGATCACCACGTTCC |  |
| *F3H*  (unigene11362) | pF: CAGGGATGATGGGAAGACG | 205 |
|  | pR: AGCCTGCTGCTGTTTGAGTT |  |
| *TEF2*  (TC3544) | pF: ATGAGCAAGTCACCCAACA | 129 |
|  | pR: AACCAAACTCTTCAGCCAATA |  |
